# Supplementary material for: Mutations in PCYT2 disrupt etherlipid biosynthesis and cause a complex hereditary spastic paraplegia
Source: Brain. 2019 Oct 22;142(11):3382–97. doi: 10.1093/brain/awz291 (PMC6821184; doi:10.1093/brain/awz291)
Supplement: awz291_Supplementary_Data [file awz291_supplementary_data.zip › awz291-Suppl_data/Supplementary_Data4.zip!/Supplementary information 3 - fibroblast lipidomics.zip!/Fibroblast lipidomics/Controle vs Patient1/Statistical plots and data/CL species/Boxplots.pdf]

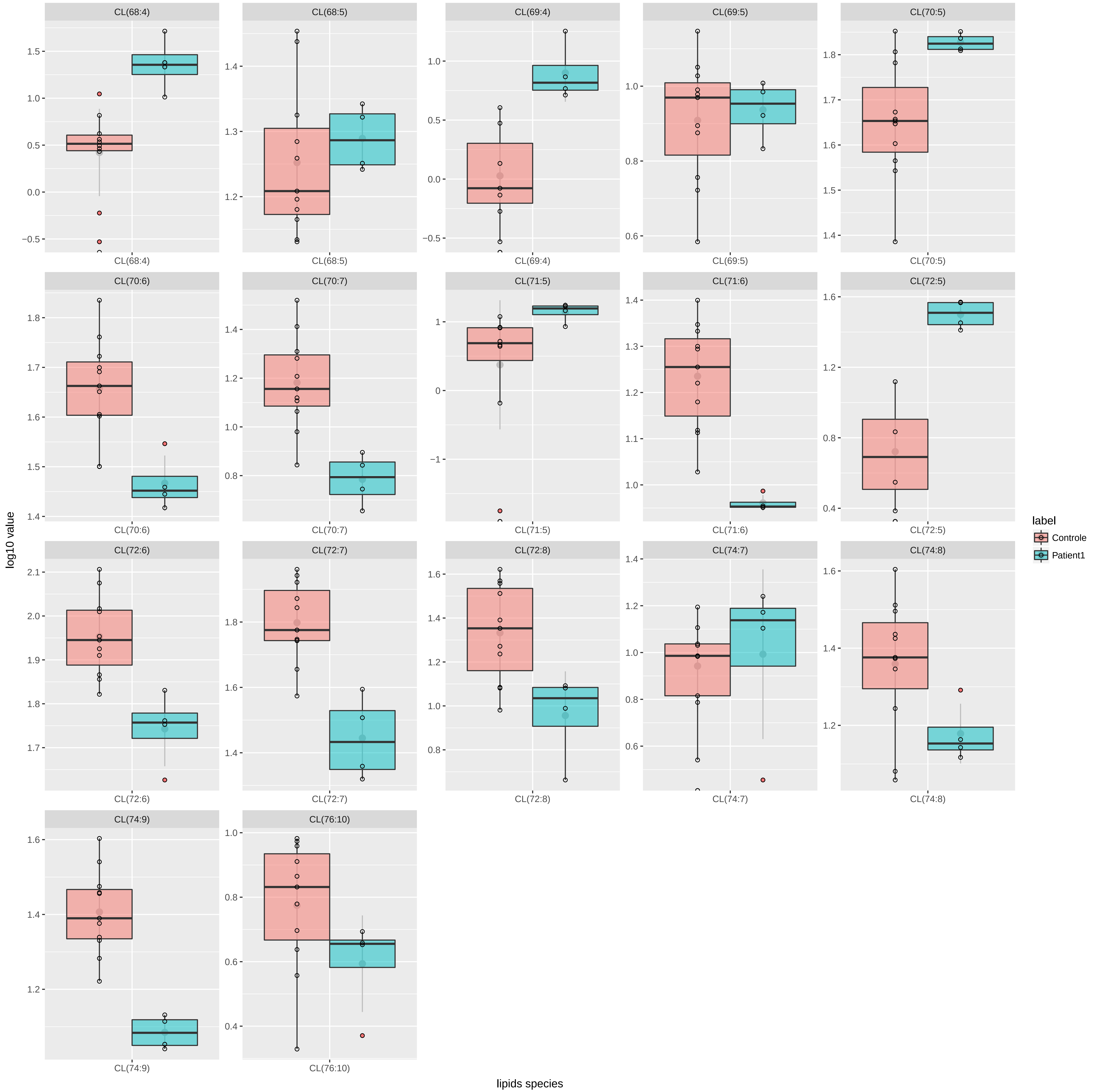

CL(68:4)
CL(68:5)
CL(69:4)
CL(69:5)
CL(70:5)
CL(70:6)
CL(70:7)
CL(71:5)
CL(71:6)
CL(72:5)
CL(72:6)
CL(72:7)
CL(72:8)
CL(74:7)
CL(74:8)
CL(74:9)
CL(76:10)
log10 value
lipids species
label
Controle
Patient1
